# Association between invasively measured central aortic pulse pressure and diameter of ascending aorta

**DOI:** 10.1038/s41598-023-48597-1

**Published:** 2023-11-30

**Authors:** Hack-Lyoung Kim, Hyun Sung Joh, Woo-Hyun Lim, Jae-Bin Seo, Sang-Hyun Kim, Joo-Hee Zo, Myung-A Kim

**Affiliations:** Division of Cardiology, Department of Internal Medicine, Boramae Medical Center, Seoul National University College of Medicine, 5 Boramae-Ro, Dongjak-Gu, Seoul, 07061 Republic of Korea

**Keywords:** Cardiology, Medical research, Pathogenesis

## Abstract

Data on the relationship between arterial pulsatile hemodynamics and aortic root geometry, using invasive hemodynamic measurement, has been scarce. Thus, this study aimed to assess the relationship between invasively measured aortic pulse pressure (aPP) and the diameter of ascending aorta (AoD). We analyzed 665 subjects (64.3 ± 11.0 years; 34.6% female) who underwent elective invasive coronary angiography (ICA) for the evaluation of coronary artery disease. Transthoracic echocardiography was performed on the same day, and AoD was measured at the level of 1 cm above the sinotubular junction at the end-diastole. Body surface area (BSA)-adjusted AoD (AoD/BSA) was used for the analysis. A pig-tail catheter was used to measure aortic pressures at a level approximately 3 cm above the aortic valve just before ICA. aPP was calculated as the difference between systolic and diastolic pressures of the aorta. In multiple linear regression analyses, aPP (*β* = 0.259; *P* < 0.001) was found to be significantly correlated with AoD/BSA even after controlling for potential confounders. This correlation power was stronger than aortic systolic pressure (*β* = 0.189; *P* < 0.001) and brachial pulse pressure (*β* = 0.091; *P* = 0.018) at the same multivariable analyses. In conclusion, our study demonstrated a significant association between invasively measured aPP and AoD/BSA, providing stronger evidence for the link between central aortic pulsatile hemodynamics and aortic root geometry.

## Introduction

Detecting aortic root dilation is crucial in clinical practice, as it is a known risk factor for serious cardiovascular conditions such as aortic regurgitation, aortic dissection, and sudden cardiac death^1^. Certain medical conditions, including aortic aneurysm, aortic dissection, and aortic regurgitation, can affect the size of the aortic root^1^, while some individuals may naturally have an enlarged aortic root^2–4^. For individuals with an enlarged aortic root, identification of risk factors that contribute to increased aortic root size is essential. This knowledge can aid in the early detection of potential cardiovascular complications and inform the development of personalized treatment plans to reduce the risk of adverse outcomes^5,6^.

Arterial pulsatile hemodynamics concerns the variations in blood flow and pressure within the arteries with each heartbeat^7^. Arterial stiffness, an aspect of arterial pulsatile hemodynamics, is characterized by the diminished capacity of arteries to expand and recoil in response to blood pressure (BP) changes. Arterial pulsatile hemodynamics can be assessed using various measurements, including pulse wave velocity (PWV), augmentation index, and pulse pressure^8^. It plays a crucial role in understanding cardiovascular disease (CVD) and mortality^9,10^. Research exploring the link between arterial pulsatile hemodynamics and aortic root size has yielded varying results. While the majority of studies indicate a positive correlation between these factors^11–16^, there are also reports of negative correlations^17,18^. It is important to note that most of these studies relied on non-invasive methods to gauge arterial pulsatile hemodynamics, which might not be as precise. In contrast, the invasive measurement of central aortic pressure provides a more accurate assessment of the heart's pressure and flow dynamics, as well as those of the major arteries^19^.

This study specifically focused on invasively measuring central aortic pressure in subjects who underwent invasive coronary angiography (ICA), and investigated the association between central aortic pulse pressure (aPP) and the diameter of the ascending aorta (AoD) measured by echocardiography on the same day. By using invasive measures, this study aims to provide more precise information about the relationship between central artery hemodynamics and aortic root size.

## Methods

### Study subjects

This cross-sectional study enrolled subjects who underwent ICA for the evaluation of coronary artery disease (CAD) at a general hospital located in Seoul, Republic of Korea. Transthoracic echocardiography (TTE) was performed on the same day before ICA. All patients were hemodynamically stable and did not report chest pain at rest. No significant changes in treatment occurred at the time of ICA after TTE. Between March 2015 and March 2018, a total of 1,074 subjects were initially screened, and 409 were excluded due to the following conditions: (1) left ventricular (LV) ejection fraction < 50%, (2) regional wall motion abnormality, (3) significant valvular dysfunction greater than mild degree, (4) congenital heart disease, (5) pericardial effusion, and (6) unreliable measurement of AoD due to poor visualization. After these exclusions, a total of 665 subjects were finally analyzed in this study. The study protocol was approved by the institutional review board (IRB) of Boramae Medical Center (Seoul, Republic of Korea), and written informed consent was obtained from each study subject.

### Clinical data collection

Height and weight were measured just before TTE, and body mass index (BMI) was calculated by dividing weight (kg) by the square of height (m^2^). Body surface area (BSA) was calculated using the following formula: BSA (m^2^) = [height (cm) × weight (kg)/3600]^½^. Hypertension was diagnosed based on the previous diagnosis by a physician, current use of anti-hypertensive medications for BP control, or systolic BP ≥ 140 mmHg and/or diastolic BP ≥ 90 mmHg in repeated examinations. Diabetes mellitus was diagnosed based on the previous diagnosis by a physician, current use of anti-diabetic medications, or fasting glucose ≥ 126 mg/dL and/or glycated hemoglobin (HbA1c) ≥ 6.5%. Dyslipidemia was diagnosed based on the previous diagnosis by a physician, current use of anti-dyslipidemic medications, or low-density lipoprotein (LDL) cholesterol level ≥ 160 mg/dL. Previous CAD included myocardial infarction and coronary revascularization. After 12 h of overnight fasting, venous blood was drawn from the antecubital vein, and the following parameters were measured: white blood cell count, hemoglobin, glucose, HbA1c, creatinine, total cholesterol, LDL cholesterol, high-density lipoprotein cholesterol, triglycerides, and C-reactive protein. The estimated glomerular filtration rate (GFR) was calculated using the Modification of Diet in Renal Disease (MDRD) equation. Information on vasoactive medications, such as renin-angiotensin system blockers, calcium-channel blockers, and statins, was also collected.

### TTE and the measurement of AoD

**Figure 1:**
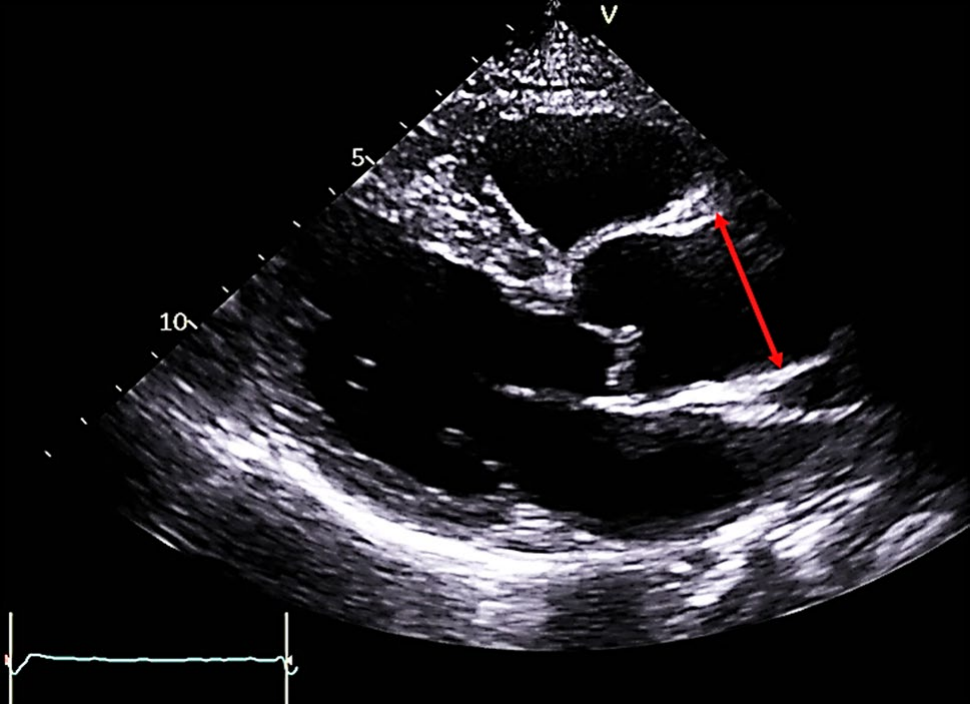
The measurement of the size of ascending aorta by transthoracic echocardiography. The measurement is taken at end-diastole in the parasternal long-axis view, with the size indicated by a red arrow.

TTE was performed before ICA on the same day. There were no medication changes and new procedures between TTE and ICA. TTE was performed with a phased array 2.5-MHz probe using commercially available equipment (EPIQ 7 and EPIQ CVx; Philips Ultrasound, Inc., Bothell, WA, USA or Vivid E9 and Vivid E95; GE Healthcare, Horten, Norway). LV ejection fraction was measured using Simpson’s biplane method. Septal e′ velocity was obtained using tissue Doppler imaging, and E/e′ was calculated. Left atrial volume index was measured using the biplane method. Maximal velocity of tricuspid regurgitation flow was measured in a modified four-chamber view. In parasternal long-axis view, measurements of maximum AoD was taken perpendicular to the long axis of the aorta, at the level of 1 cm above the sinotubular junction at the end-diastole^20^. Measurements were taken using the leading edge-to-leading edge technique (Fig. 1)^21^. When the aorta was not well-visualized on the standard view, an intercostal space was raised to obtain an image.

### Noninvasive measurement of brachial BP

Immediately before TTE, the patient’s brachial BP was measured in the upper right arm using an automatic oscillometric device. The patient was instructed to refrain from smoking, drinking caffeine, or exercising for at least 30 min prior to the BP measurement. Additionally, the patient was advised to sit quietly for several minutes prior to the measurement to allow their body to relax and stabilize their BPs. Systolic and diastolic BPs were measured, and brachial pulse pressure (bPP) was calculated as the difference between systolic and diastolic BPs. Brachial BPs were measured several times at 1–2 min intervals, and the average of at least two values was used^22^.

### Invasive measurement of aortic BP

The ICA procedure was performed using either a radial or femoral approach. Aortic pressure measurement was performed just before the ICA procedure. Under local anesthesia at the puncture site, a 5 French pigtail catheter was inserted into the ascending aorta. The aortic BP was measured at a level approximately 3 cm above the aortic valve^23^, which corresponds to the location where AoD is measured using TTE. Systolic and diastolic BPs were measured, and aPP was calculated as the difference between systolic and diastolic BPs. Aortic pressure curves from at least five cardiac cycles were recorded, and the value was determined by averaging three consecutive cycles. After the aPP measurement was taken, the ICA procedure was performed according to standardized recommendations.

### Statistical analysis

Continuous variables are expressed as mean ± standard deviation, while discrete variables are expressed as percentages. Pearson’s correlation analysis was used to assess simple linear correlation between the two variables. Multiple linear regression analysis was performed to assess the independent association between BP profiles and AoD normalized by BSA (= AoD/BSA), while adjusting for covariates including age, sex, heart rate, HbA1c, LDL cholesterol, and estimated GFR. These independent variables are known to be related to aortic size, and given that this is a linear analysis, we aimed to select continuous variables as much as possible while avoiding multicollinearity. Multicollinearity was assessed using the variance inflation factor, with the value being < 2, which indicates the absence of multicollinearity. Scatter plots were used to demonstrate the associations of bPP and aPP with AoD/BSA. Statistical significance was set at a *P* value of < 0.05. All statistical analyses were conducted using SPSS for Windows version 22 (IBM Co., Armonk, NY, USA).

### Ethics approval

This study was performed in line with the principles of the Declaration of Helsinki. Approval was granted by the Ethics Committee of Boramae Medical Center (Date: 2015-02-25/No: 16-2015-24).

### Consent to participate

Informed consent was obtained from all individual participants included in the study.

## Results

### Clinical characteristics of study subjects

Clinical characteristics of study participants are presented in Table 1. The mean age of study subjects was 64.3 ± 11.0 years and 34.6% of them were women. The prevalence of hypertension, diabetes mellitus, dyslipidemia and cigarette smoking were 66.3%, 32.3%, 37.7% and 26.8%, respectively. Almost half of the study subjects (45.0%) had a previous history of CAD, and 2.7% had a history of stroke. Stable angina pectoris was the most common diagnosis (86.3%) at the time of ICA. Most of major blood tests and echocardiographic parameters were within normal ranges. The majority of the study participants were taking renin-angiotensin system blockers (78.0%), beta-blockers (72.9%) and statins (95.9%), while about half of them were taking calcium-channel blockers (49.6%).

**Table 1:** Clinical characteristics of study participants.

Characteristic	Value (n = 665)
Age (years)	64.3 ± 11.0
Female sex	230 (34.6)
Height (cm)	161 ± 9
Weight (kg)	65.6 ± 11.8
Body mass index (kg/m^2^)	25.2 ± 3.4
Body surface area (m^2^)	1.71 ± 0.18
Cardiovascular risk factors	
Hypertension	441 (66.3)
Diabetes mellitus	215 (32.3)
Dyslipidemia	251 (37.7)
Current smoking	178 (26.8)
Obesity (body mass index ≥ 25 kg/m^2^)	345 (51.9)
Previous coronary artery disease	299 (45.0)
Previous stroke	18 (2.7)
Clinical diagnosis	
Stable angina	574 (86.3)
Acute coronary syndrome	43 (6.5)
Others	48 (7.2)
Laboratory findings	
White blood cell count (per µL)	6873 ± 1959
Hemoglobin (g/dL)	13.1 ± 1.6
Glucose (mg/dL)	116 ± 37
Glycated hemoglobin (%)	6.54 ± 1.42
Estimated glomerular filtration rate (mL/min/1.73 m^2^)	82.7 ± 23.8
Total cholesterol (mg/dL)	147 ± 37
Low-density lipoprotein cholesterol (mg/dL)	85.3 ± 33.4
High-density lipoprotein cholesterol (mg/dL)	43.4 ± 11.7
Triglyceride (mg/dL)	123 ± 76
C-reactive protein (mg/dL)	0.65 ± 2.08
Echocardiographic findings	
Left ventricular ejection fraction (%)	65.3 ± 5.9
Septal eʹ velocity (cm/s)	6.18 ± 1.82
E/eʹ	11.2 ± 4.6
Left atrial volume index (mL/m^2^)	31.3 ± 10.9
Maximal velocity of tricuspid regurgitation (m/s)	2.32 ± 0.36
Cardiovascular medications	
Renin-angiotensin system blockers	519 (78.0)
Calcium-channel blockers	330 (49.6)
Beta-blockers	485 (72.9)
Statins	638 (95.9)

Numbers are expressed as mean ± standard deviation or n (%).

BP profiles and heart rate are shown in Table 2. Mean systolic BP noninvasively measured at right brachial artery was 129 ± 15 mmHg and mean systolic BP invasively measured at aorta was 139 ± 21 mmHg. The mean values of bPP and aPP were 52.0 ± 11.3 and 67.4 ± 18.6, respectively. Mean values of AoD and AoD/BSA were 33.9 ± 3.7 cm and 19.8 ± 3.4 cm/m^2^, respectively.

**Table 2:** BP profiles and heart rate.

BP parameter	Value (n = 665)
Brachial systolic BP	129 ± 15
Brachial diastolic BP	77.6 ± 10.0
Brachial pulse pressure	52.0 ± 11.3
Heart rate, per minute	65.8 ± 11.9
Aortic systolic BP	139 ± 21
Aortic diastolic BP	68.6 ± 15.0
Aortic pulse pressure	67.4 ± 18.6

*BP* blood pressure.

### Association between BP profiles and AoD

**Figure 2:**
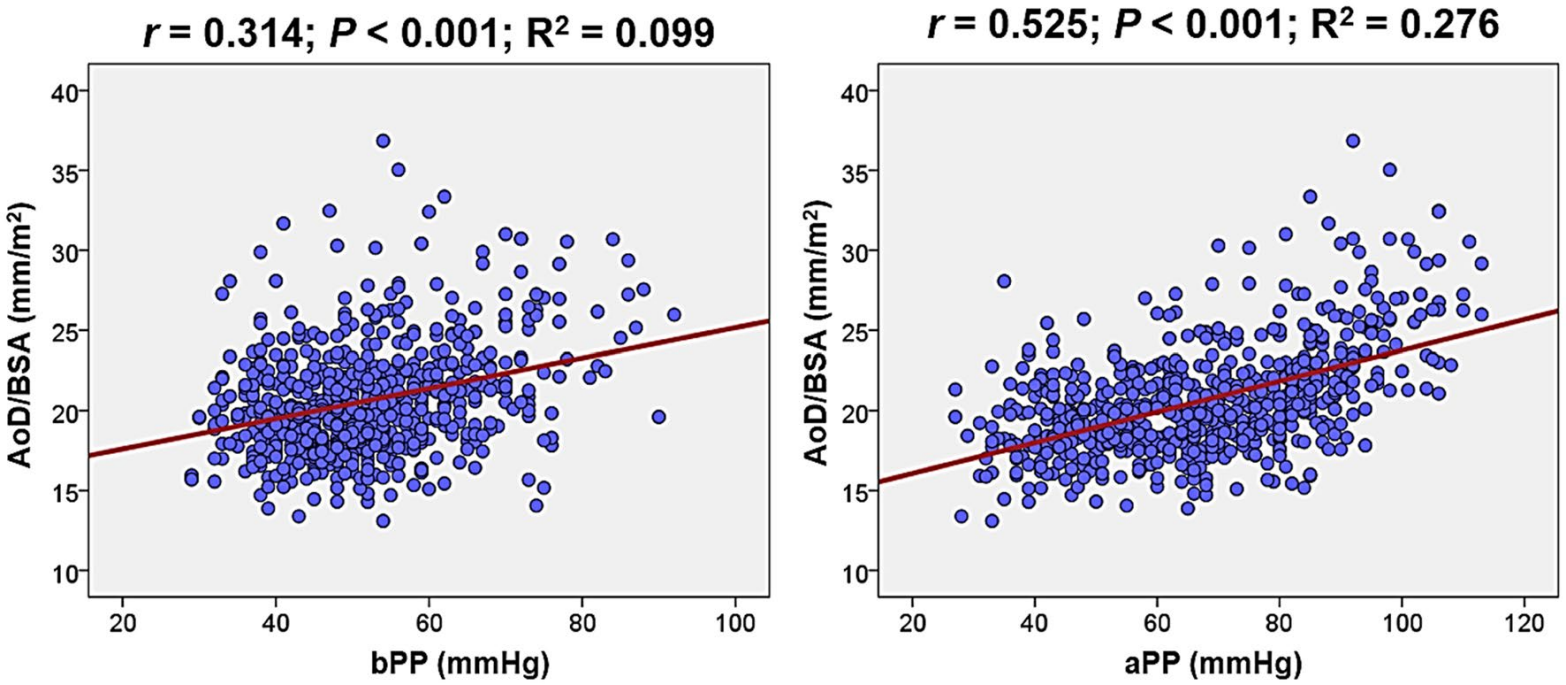
Scatter plots showing the associations of AoD/BSA with bPP and aPP. *AoD* ascending aorta diameter, *BSA* body surface area, *bPP* brachial pulse pressure, *aPP* aortic pulse pressure.

In simple linear correlations (Table 3), there was a positive correlation between brachial systolic BP (*r* = 0.136; *P* < 0.001) and bPP (*r* = 0.314; *P* < 0.001) with AoD/BSA, while brachial diastolic BP (*r* = − 0.124; *P* = 0.001) was negatively correlated with AoD/BSA. AoD/BSA was positively correlated with aortic systolic BP (*r* = 0.349; *P* < 0.001) and aPP (*r* = 0.525; *P* < 0.001) and negatively correlated with aortic diastolic BP (*r* = − 0.218; *P* < 0.001). The correlation was stronger for PP than for systolic BP, both for brachial and aortic pressures. Additionally, the correlation between aortic pressures and AoD/BSA was stronger than the correlation between brachial pressures and AoD/BSA. The strongest correlation was observed between aPP and AoD/BSA. The correlations of AoD/BSA with bPP and aPP are shown as scatter plots in Fig. 2. In multiple linear regression analyses (Table 4), bPP (*β* = 0.091; *P* = 0.018), aortic systolic BP (*β* = 0.189; *P* < 0.001) and aPP (*β* = 0.259; *P* < 0.001) remained significantly correlated with AoD/BSA even after controlling for potential confounders. The strongest correlation was observed between aPP and AoD/BSA. The correlation strength was somewhat weaker when analyzed with AoD/BMI compared to AoD/BSA, yet the trend of a stronger correlation with aPP compared to bPP persisted (Supplementary Table S1). In the multivariable analysis models, even after adjusting for potential confounders, older age and female sex were significantly associated with a larger AoD/BSA, in addition to BP indices (Supplementary Table S2).

**Table 3:** Simple correlation between each BP parameter and AoD/BSA.

BP parameter	*r*	*P*
Brachial systolic BP	0.136	< 0.001
Brachial diastolic BP	− 0.124	0.001
Brachial pulse pressure	0.314	< 0.001
Aortic systolic BP	0.349	< 0.001
Aortic diastolic BP	− 0.218	< 0.001
Aortic pulse pressure	0.525	< 0.001

*BP* blood pressure, *AoD* the diameter of ascending aorta, *BSA* body surface area.

**Table 4:** Multiple linear regression analysis showing the independent associations of BP parameters and AoD/BSA.

Variable	*β*	t	*P*
Brachial systolic BP	0.033	0.916	0.360
Brachial diastolic BP	− 0.033	− 0.936	0.350
Brachial pulse pressure	0.091	2.374	0.018
Aortic systolic BP	0.189	5.382	< 0.001
Aortic diastolic BP	0.018	0.476	0.634
Aortic pulse pressure	0.259	6.552	< 0.001

Separate multivariable analysis was performed for each variable. The following covariates were adjusted: age, sex, heart rate, glycated hemoglobin, low-density lipoprotein cholesterol, and estimated glomerular filtration rate.

*BP* blood pressure, *AoD* the diameter of ascending aorta, *BSA* body surface area.

## Discussion

Our study demonstrated that AoD/BSA was significantly correlated with both bPP and aPP in multivariable analysis of subjects who underwent ICA. However, the correlation was stronger in aPP than in bPP. Our study, utilizing invasive measurements, provides stronger evidence of the association between central aortic pulsatile hemodynamics and aortic root size.

Several studies have suggested that there is an association between aortic root geometry and arterial pulsatile hemodynamics. Most of the studies that analyzed this issue targeted patients with pathological conditions such as aortic dilatation or dissection, and the number of analyzed patients was small. In a study that analyzed 33 patients undergoing aortic replacement surgery for aortic dilatation, it was confirmed that the distensibility of the aorta measured by ultrasound was lower than that of the control group^24^. In another study, the PWV of the aorta of 40 patients with thoracic aortic aneurysm was measured using magnetic resonance imaging, and the authors showed that the local PWV was increased at the site of aortic dilatation^25^. The association between arterial pulsatile hemodynamics and aortic root geometry was also reported in patients with bicuspid aortic valve^26^, tetralogy of Fallot^27^ and single ventricular circulation^28^. On the other hand, studies examining the relationship between arterial pulsatile hemodynamics and aortic size in subjects without significant structural problems in the aorta or heart are relatively rare. Previously, our group have shown that BSA corrected aortic root diameters including sinotubular junction/BSA and AoD/BSA were well correlated with brachial-ankle PWV in women but not in men among 263 subjects had no CVD^11^. Virz et al*.*^16^ measured both aortic root diameters and arterial stiffness using echocardiography in 422 subjects without CVD. They showed that while the aortic diameter increased with age, but the extent of this increase decreased when correcting for arterial stiffness. This suggests that arterial stiffness may be an important contributor to the age-related increase in aortic size. In another study, 345 hypertensive patients were analyzed, and it was found that arterial stiffness measured by tonometry was correlated with proximal aortic diameter measured by echocardiography^14^. Totaro et al.^15^ investigated 177 young subjects (11–35 years), and demonstrated that aPP measured using noninvasive arterial tonometry was an independent predictor for the dilatation of proximal aorta. Similarly, in another study of 190 untreated hypertensive subjects, aPP was measured using tonometry, and an independent association between aortic root dilation and aPP was shown^13^. These findings suggest a positive association between arterial pulsatile hemodynamics and aortic root diameter, which is in consistent with our study result. However, in contrast to these previous studies, our study has the strength of using an invasive method to measure aPP, and we also enrolled larger number of subjects. A European study, similar to ours, invasively measured aPP in patients undergoing ICA and found a negative correlation between aPP and the z-score of the sinus of Valsalva of the aorta^18^. These differences in research outcomes may be attributed to racial differences, variations in research methods, and other factors. However, our results can be considered more robust because our analyzed patient cohort was significantly larger (n = 665) than that study (n = 71). Future research is expected to provide more precise insights.

Compared to peripheral arterial pressure, central arterial pressure is expected to exert a greater influence on major organs due to its proximity^19^. Many studies have suggested that central aortic BP is better associated with target organ damage and clinical outcomes than brachial BP^29–31^. Milan et al.^13^ measured central BP using tonometry in 190 hypertensive patients and showed that aPP was more closely associated with the dilation of ascending aorta than bPP. Consistent with these findings, our study showed a stronger association between invasively measured aPP and AoD/BSA than bPP.

Both arterial stiffness and aortic root geometry are important predictors of CVD and mortality. Therefore, understanding the relationship between these two measures is crucial, as it could help identify individuals who are at higher risk for developing these conditions and allow for earlier interventions. However, as described above, most previous studies on this issue have measured aortic pressure using non-invasive methods. Considering that invasive hemodynamic study is the gold standard method ^19^, our study results deserve attention by providing stronger evidence on the relationship between arterial pulsatile hemodynamics and aortic diameter. Additionally, we demonstrated that aPP was more strongly associated with AoD/BSA than bPP, highlighting the importance of measuring central artery pressure. Furthermore, understanding the association between arterial pulsatile hemodynamics and aortic root geometry could have important implications for the development of new treatment strategy. For example, interventions that improve arterial stiffness may also have a favorable influence on aortic root geometry, potentially reducing the risk of cardiovascular events.

Our results demonstrated that the systolic and pulse pressures in the central artery were higher than those in the brachial artery. It is generally observed that arterial stiffness increases from central to peripheral regions, causing pulse pressure amplification and, consequently, higher systolic blood pressure or pulse pressure in central arteries compared to peripheral arteries^19^. However, this trend often reverses in elderly and high-risk patients. As individuals age or are exposed to risk factors over extended periods, the escalation in arterial stiffness is more severe in central arteries, rich in elastic fibers, than in peripheral arteries. This leads to an inversion of this pressure disparity^32–34^. Given that our study included older individuals, with a mean age of 64 years, and high-risk patients undergoing invasive coronary angiography, the presence of this phenomenon aligns with expectations.

In our study, AoD/BSA showed a negative correlation with diastolic BP in the univariate analysis, although this significance was not observed in the multivariate analysis. As arteries age or are subjected to risk factors for extended periods, they undergo a remodeling process, resulting in arterial enlargement and increased stiffness^35^. Such increased stiffness contributes to elevated systolic or pulse pressure and reduced diastolic BP^36^.

### Study limitations

There are several limitations to our study. First, this is a cross-sectional study, and thus we could not confirm the causal relationship between aPP and AoD/BSA. To confirm the causal relationship, a longitudinal study that serially analyzes AoD changes according to changes in arterial stiffness is needed. Second, there is a time difference of several hours between the AoD and aPP measurements, even though they were performed on the same day. However, we followed the order of measuring AoD first and then measuring aPP, and no other drugs or procedures were added between the two measurements. Third, our study utilized a variety of echocardiography machines. However, we did not account for the potential variability between these different devices. Lastly, caution should be exercised in generalizing the results of our study since it involved patients undergoing ICA.

## Conclusions

Our study found that invasively measured aPP was more strongly associated with AoD/BSA than noninvasively measured bPP in patients who underwent ICA. This provides stronger evidence on the association between central arterial pulsatile hemodynamics and aortic root geometry.

### Supplementary Information

Supplementary Tables.

## Data Availability

All data generated or analyzed during this study are included in this article.
