# Supplementary material for: Association between invasively measured central aortic pulse pressure and diameter of ascending aorta
Source: Sci Rep. 2023 Nov 30;13:21152. doi: 10.1038/s41598-023-48597-1 (PMC10689808; doi:10.1038/s41598-023-48597-1)
Supplement: Supplementary file 1 — Supplementary Tables. [file 41598_2023_48597_MOESM1_ESM.docx]

**Supplementary data**

**Supplementary Table S1. Multiple linear regression analysis showing the independent associations of BP parameters and AoD/BMI**

| **Variable** | ***β*** | **t** | ***P*** |
| --- | --- | --- | --- |
| Brachial systolic BP | 0.042 | 1.041 | 0.254 |
| Brachial diastolic BP | -0.017 | -0.750 | 0.418 |
| Brachial pulse pressure | 0.102 | 2.724 | 0.025 |
| Aortic systolic BP | 0.145 | 3.102 | < 0.001 |
| Aortic diastolic BP | 0.014 | 0.605 | 0.653 |
| Aortic pulse pressure | 0.190 | 3.980 | < 0.001 |

Separate multivariable analysis was performed for each variable. The following covariates were adjusted: age, sex, heart rate, glycated hemoglobin, low-density lipoprotein cholesterol, and estimated glomerular filtration rate. BP, blood pressure; AoD, the diameter of ascending aorta; BMI, body mass index.

**Supplementary Table S2. Multiple linear regression analysis showing the independent associations of BP parameters and AoD/BSA**

| **Variable** | ***β*** | **t** | ***P*** | **VIF** |
| --- | --- | --- | --- | --- |
| *Model with brachial systolic BP as an independent variable* | | | | |
| Age | 0.491 | 12.785 | < 0.001 | 1.258 |
| Sex | 0.331 | 9.207 | < 0.001 | 1.101 |
| Heart rate | 0.005 | 0.148 | 0.883 | 1.098 |
| Glycated hemoglobin | -0.029 | -0.820 | 0.413 | 1.096 |
| LDL cholesterol | -0.016 | -0.444 | 0.657 | 1.051 |
| Estimated GFR | -0.005 | -0.144 | 0.885 | 1.195 |
| Brachial systolic BP | 0.033 | 0.916 | 0.360 | 1.091 |
| *Model with brachial diastolic BP as an independent variable* | | | | |
| Age | 0.492 | 12.804 | < 0.001 | 1.256 |
| Sex | 0.331 | 9.204 | < 0.001 | 1.101 |
| Heart rate | 0.009 | 0.249 | 0.804 | 1.086 |
| Glycated hemoglobin | -0.018 | -0.504 | 0.615 | 1.074 |
| LDL cholesterol | -0.019 | -0.530 | 0.596 | 1.186 |
| Estimated GFR | -0.008 | -0.222 | 0.825 | 1.186 |
| Brachial diastolic BP | -0.033 | -0.936 | 0.350 | 1.061 |
| *Model with brachial pulse pressure as an independent variable* | | | | |
| Age | 0.468 | 11.772 | < 0.001 | 1.359 |
| Sex | 0.321 | 8.195 | < 0.001 | 1.118 |
| Heart rate | 0.009 | 0.241 | 0.810 | 1.081 |
| Glycated hemoglobin | -0.038 | -1.060 | 0.290 | 1.085 |
| LDL cholesterol | -0.012 | -0.345 | 0.730 | 1.052 |
| Estimated GFR | 0.004 | 0.097 | 0.923 | 1.208 |
| Brachial pulse pressure | 0.091 | 2.374 | 0.018 | 1.254 |
| *Model with aortic systolic BP as an independent variable* | | | | |
| Age | 0.475 | 12.738 | < 0.001 | 1.253 |
| Sex | 0.293 | 8.211 | < 0.001 | 1.148 |
| Heart rate | 0.003 | 1.00 | 0.920 | 1.080 |
| Glycated hemoglobin | -0.023 | -0.685 | 0.494 | 1.051 |
| LDL cholesterol | -0.012 | -0.345 | 0.730 | 1.048 |
| Estimated GFR | 0.013 | 0.368 | 0.713 | 1.201 |
| Aortic systolic BP | 0.189 | 5.382 | < 0.001 | 1.108 |
| *Model with aortic diastolic BP as an independent variable* | | | | |
| Age | 0.503 | 12.161 | < 0.001 | 1.458 |
| Sex | 0.333 | 9.267 | < 0.001 | 1.098 |
| Heart rate | 0.004 | 0.125 | 0.901 | 1.093 |
| Glycated hemoglobin | -0.021 | -0.589 | 0.556 | 1.064 |
| LDL cholesterol | -0.017 | -0.495 | 0.621 | 1.048 |
| Estimated GFR | -0.009 | 0.237 | 0.813 | 1.187 |
| Aortic diastolic BP | 0.018 | 0.476 | 0.634 | 1.250 |
| *Model with aortic pulse pressure as an independent variable* | | | | |
| Age | 0.399 | 10.131 | < 0.001 | 1.440 |
| Sex | 0.270 | 7.571 | < 0.001 | 1.182 |
| Heart rate | 0.018 | 0.512 | 0.609 | 1.083 |
| Glycated hemoglobin | -0.040 | -1.175 | 0.241 | 1.057 |
| LDL cholesterol | -0.011 | -0.319 | 0.750 | 1.048 |
| Estimated GFR | 0.029 | 0.800 | 0.424 | 1.216 |
| Aortic pulse pressure | 0.259 | 6.552 | < 0.001 | 1.450 |

BP, blood pressure; AoD, the diameter of ascending aorta; BSA, body surface area; VIF, variance inflation factors; LDL, low-density lipoprotein; GFR, glomerular filtration rate.
